# Genomic Insights into Date Palm Origins

**DOI:** 10.3390/genes9100502

**Published:** 2018-10-17

**Authors:** Muriel Gros-Balthazard, Khaled Michel Hazzouri, Jonathan Mark Flowers

**Affiliations:** 1 Center for Genomics and Systems Biology, New York University Abu Dhabi, Saadiyat Island, P.O. Box 129188, Abu Dhabi, UAE; jmf11@nyu.edu; 2 Khalifa Center for Genetic Engineering and Biotechnology (KCGEB), United Arab Emirates University, P.O. Box 15551, Al Ain, UAE; khaled_hazzouri@uaeu.ac.ae; 3 Department of Biology, Center for Genomics and Systems Biology, 12 Waverly Place, New York University, New York, NY 10003, USA

**Keywords:** *Phoenix dactylifera*, domestication, next-generation sequencing, population genetics

## Abstract

With the development of next-generation sequencing technology, the amount of date palm (*Phoenix dactylifera* L.) genomic data has grown rapidly and yielded new insights into this species and its origins. Here, we review advances in understanding of the evolutionary history of the date palm, with a particular emphasis on what has been learned from the analysis of genomic data. We first record current genomic resources available for date palm including genome assemblies and resequencing data. We discuss new insights into its domestication and diversification history based on these improved genomic resources. We further report recent discoveries such as the existence of wild ancestral populations in remote locations of Oman and high differentiation between African and Middle Eastern populations. While genomic data are consistent with the view that domestication took place in the Gulf region, they suggest that the process was more complex involving multiple gene pools and possibly a secondary domestication. Many questions remain unanswered, especially regarding the genetic architecture of domestication and diversification. We provide a road map to future studies that will further clarify the domestication history of this iconic crop.

## Highlights

- A large amount of genomic data has been generated for the date palm in the past few years.
- This review records these data and re-evaluates questions about date palm domestication origins and diffusion in light of genomic data
- Remaining questions are highlighted, and the future perspective of the field discussed.

## 1. Introduction

Plant domestication accompanying the development of agriculture is a significant step in human history. The understanding of the origins of crops is an important area of research involving scientists from many disciplines such as archaeology, anthropology, and, more recently, genetics and genomics. The main questions relate to where and when the plant was domesticated and what progenitor populations were involved. The molecular and phenotypic changes associated with domestication are also of prime interest. With the advent of next-generation sequencing (NGS) technologies, new advances have been made at an unprecedented pace. The availability of whole genome sequence data has indeed led to answers to many questions related to crop evolution. Such answers include a refinement of geographic origins (e.g., in African rice [1]) and the identification of genes controlling agronomically important traits (e.g., in maize [2]). This progress contributes to the awareness of human history and provides clues to breeders who are developing tomorrow’s crops in the context of global change [3,4]. 

**Figure 1:**
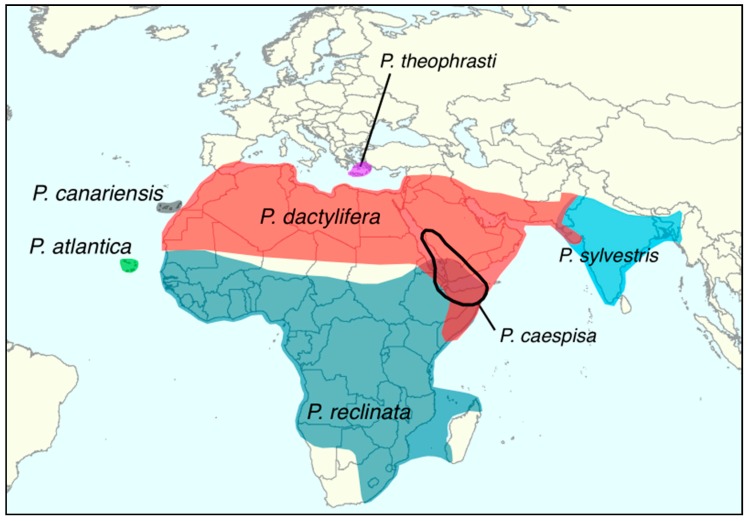
Distribution of the date palm and *Phoenix* species in Europe, Africa and Western Asia [5,6].

The date palm is an iconic crop of hot and arid regions of North Africa and the Middle East, Figure 1. It is a member of genus *Phoenix* which consists of 14 perennial and dioecious monocotyledonous species in the Arecaceae family [5]. 

The namesake fruit of the date palm can be eaten fresh or dried or transformed into a large variety of products such as syrup or paste. The sweet fruit has played a prominent role in nomadic societies of desert regions and is firmly rooted in Arabic culture. Each year, more than eight million tons are produced worldwide and this number is constantly growing owing to large scale efforts to increase the numbers of fruit-producing trees [7]. The date palm is also a key species of oasian agrosystems as virtually every part of the plant finds a particular use in daily life [5]: the stipe (or trunk) is used for construction, the leaves for basketry, and more importantly, the date palm provides a favorable microclimate for the culture of other crops in an otherwise very harsh environment [8]. In cultivation, this species is reproduced through a mixed clonal-sexual system although clonal propagation is preferred in most circumstances to maintain fruit quality [9]. Approximately 3000 cultivars are believed to exist [10], however this number is extremely difficult to assess. Indeed, cultivars are supposedly clones bearing the same name, but cases of homonymy have been reported (e.g., in Morocco [11]) and, conversely, individuals bearing the same name without being clones have been identified (e.g., in Egypt [12]), making it difficult to count and inventory date palm cultivars.

The history of domestication and diversification of the date palm has remained a puzzling question despite decades of research from different disciplines. The identity of its wild progenitor has long been unknown and has hampered research into its origins [13]. The first archaeological records of exploitation and consumption of dates are from the 6th millennium BCE [8]. There is evidence of cultivation in the Gulf region since the late 4th/early 3rd millennium BCE while seeming to appear later in Africa, although this should be confirmed by further studies [8]. Many developments in the understanding of the date palm domestication history have been made in the past decade based on genetics and later genomics as the genome sequence became available and resequencing studies have been carried out. In this paper, we review the genomic data available for the date palm and highlight the recent progresses made from this data on the question of origins. Further, we report the still remaining questions and propose a guideline to elucidate them.

## 2. High-Throughput Sequencing of Date Palms 

Next-generation sequencing refers to the numerous high-throughput alternatives to Sanger sequencing—the favorite sequencing method used since 1977—that were developed within the last decade and remain under active development [14]. These methods, and so-called 3rd generation technologies [15], enable the generation of genome assemblies (i.e., the haploid genome sequence of an organism) through de novo sequencing. Further resequencing studies allow the characterization of variation (e.g., single nucleotide polymorphisms (SNPs)) among individuals, populations, and species on a genome-wide scale. Although whole-genome resequencing is the most comprehensive approach, other experimental designs can identify polymorphisms at a lower cost through the study of a fraction of the genome. Examples include transcriptome sequencing, which targets only coding regions by sequencing messenger RNAs, and genotyping-by-sequencing methods that focus on pre-established sets of genome positions. The relatively low cost and the sequencing speed of NGS technologies have led to the accumulation of large amounts of genomic data. Along with these advantages come a number of important issues: biases related to the short sequence reads, high error rates, and problems with handling such large datasets [14]. Still, the availability of genomic data for crops represent an amazing opportunity to understand their biology and evolution.

The first crop genome to be sequenced was the rice genome [16,17] and since then many others have been made available including complex genomes such as that of wheat [18]. In this race for genomic data, the date palm is no exception. In 2009, only ~100 kbp of nuclear date palm DNA sequence was available in GenBank, an open access sequence database [19]. Since then, there have been two published efforts to sequence and assemble the date palm genome and several projects to *de novo* assemble the plastid genomes, Table 1. 

Additional studies have conducted whole genome resequencing of numerous cultivars and wild relatives (see Table 2) or sequencing of reduced representation libraries [25,26,27,28,29,30,31]. 

Eventually, in September 2018, hundreds of gigabases of data were available in GenBank. Today, an online database for date palm genomic resources has been generated with the positions of polymorphisms and microsatellite loci among 62 cultivars [32] providing information regarding cultivar identification and classification [35].

### 2.1. Nuclear Reference Genome

The date palm nuclear genome size is ~670 Mb distributed on 18 chromosomes [20,36]. A first draft (cultivar Khalas from Saudi Arabia), generated de novo, was published in 2011 [19], Table 1. At this time, it constituted the only reference genome of the order Arecales and the family Arecaceae. The 380 Mb sequence covered 60% of the nuclear genome and was mainly composed by gene-rich regions as it was predicted to contain approximately 90% of the genes and more than 25,000 gene models. This reference genome is highly fragmented with about 60,000 scaffolds—genomic sequences—showing a median length of ~30 kb. Another genome assembly of higher quality, of the same cultivar, was released in 2013 [20]. This assembly has a total length of ~600 Mb, covering more than 90% of the genome and is estimated to contain more than 96% of the genes (~41,660 genes). Nevertheless, it is still highly fragmented (>80,000 scaffolds, scaffold N50 = ~330 kb), partly because of the highly heterozygous nature of the date palm genome which is problematic for traditional haploid assembly algorithms. The Al-Mssallem et al. assembly [20] has been included in the Reference Sequence (RefSeq) collection at the National Center for Biotechnology Information (NCBI) which offers structural annotations produced by the NCBI automated pipeline, a browser to explore the genome interactively, a Basic Local Alignment Search Tool (BLAST) utility, and many other tools for genome analysis. In addition, RefSeq gene models for date palm have been included in UniProtKB (https://www.uniprot.org/) and the Kyoto Encyclopedia of Genes and Genomes (KEGG; https://www.genome.jp/kegg/). These databases provide many additional avenues for functional exploration of the genome including Gene Ontology (GO) terms and analysis of genes in the context of biochemical pathways. Although only a female cultivar has been de novo assembled, the full-length sequencing of male-specific sequences from a single male individual using PacBio and 10X Genomics technologies was recently published [34]. This provides an assembly of the *Phoenix* sex-determination region and is therefore valuable for studies of the male specific sequences.

Future prospects for date palm genomics include the generation of a better assembly through improving the junctions between scaffolds and assigning and ordering them on the 18 chromosomes. Application of recent advances in genomics including third generation (long read) sequencing technologies such as PacBio, proximity ligation sequencing technologies (Hi-C), and diploid assembly algorithms [37] should contribute to a dramatically improved assembly. Additionally, although two assemblies are annotated, and a genetic map has been generated [38], both careful structural and functional annotations are required in order to move forward in the understanding of the genome structure. Incorporation of RNA-sequencing (RNA-seq) data from additional tissues will lead to improved gene models. A finer genetic map would be valuable both for improved assemblies and for evolutionary genetic analysis. In addition, a number of approaches now in widespread use in other crops may lead to important advances in date palm genomics. For example, surveys of epigenomic variation have the prospect of opening entirely new areas of inquiry such as the nature of somaclonal variation in vegetatively propagated cultivars. Besides, the application of CRISPR/Cas9 genome editing in date palm [39] could provide a means to manipulate the genome both for studies of gene function and crop improvement. 

### 2.2. Organellar Genomes

Both mitochondrial and chloroplastic date palm genomes were among the first crop organellar genomes published. A single published assembly is available for the mitochondrial genome which is 715,001 bp in length and mainly composed of noncoding sequences (93.5%) [24]. There are two other unpublished assemblies available in GenBank from an unknown cultivar and from Khanezi, Table 1. Several assemblies are published for the chloroplastic genome: cultivar Khalas from Saudi Arabia [21], cultivar Aseel from Pakistan [22], and cultivars Naghal and Khanezi from Oman [23]. Like most other angiosperms, the chloroplastic date palm genome is a circular double-stranded DNA molecule displaying a quadripartite structure with a large and a small single-copy region that are separated by two copies of inverted repeats. It has a size of ~158,000 bp and is predicted to contain approximately 130 functional genes. There are three additional unpublished chloroplastic assemblies (GenBank references: MF197494.1, FJ212316.3, and GU811709.2) that constitute an update of the two previously published assemblies. Many polymorphisms were noted among plastid sequences [22,23] as well as heteroplasmy (i.e., more than one type of organellar genome within an individual) [40]. 

### 2.3. Resequencing Projects

Following the availability of organellar and nuclear reference genomes for date palm, several groups used whole-genome sequencing to investigate the date palm and its wild relatives. For instance, several studies identified the sex determination region [19,34]. Other studies focused on date palm diversity and history [32,33] and will be described in more details in other sections of this paper.

### 2.4. Transcriptomic Data and Analyses

In date palm, several studies have generated transcriptomic data. This includes gene profiling at seven different fruit stages or in different tissues [20,25,41] or the analysis of response to salinity [28,29]. Additionally, there are many studies that focused on other species but provided date palm transcriptomic data, such as a study on oil palm [42].

### 2.5. Ancient DNA of Date Palm

Ancient DNA (aDNA) has not yet been recovered in date palm. Nevertheless, in 2008 an Israeli team published a preliminary genetic study of an ancient seed from Masada fortress (Israel) dating back to the first century before the common era (BCE) [43]. This seed had successfully germinated and was compared to three current cultivars using random amplified polymorphic DNA. Although this is not a DNA per se, the sequencing of fresh leaves from this old sample provides a glimpse into past diversity. The germination of ancient seeds could therefore provide exceptional genetic information and help to decipher the process of domestication and diffusion of this species.

## 3. The Date Palm and the Genus *Phoenix*

The date palm *Phoenix dactylifera*, although considered a tree, is an arborescent monocotyledon from the Arecaceae family [44]. The genus comprises 14 recognized species (2n = 36), see Table 3, that are distributed in the tropical and subtropical regions of the Old World, Figure 1 [45]. The date palm is the only domesticated species in the genus but the other species are also extensively used for ornamental purposes (*Phoenix canariensis* and *Phoenix roebelenii*), food (for instance *Phoenix sylvestris* sap [46]), clothing, construction, fiber, or feed for livestock [5]. 

*Phoenix* species are morphologically very close and sometimes hardly distinguishable [50]. For instance, there are very few characteristics to separate the date palm from *Phoenix theophrasti* [5] or *P. atlantica* [48]. Interspecific barriers are weak or absent and species integrity presumably has been maintained through geographical or ecological isolation. The genus *Phoenix* thus forms a species complex where all species can freely hybridize [51]. In the Canary Islands, hybrids between *P. dactylifera* and *P. canariensis* have been reported [52,53]. In Senegal, hybrids between the sub-Saharan *Phoenix reclinata* and the date palm have been noted [51]. However, those sympatric zones where *Phoenix* hybridize are artificial and due to human-mediated spread of date palms and its wild relatives outside of their natural range. Although it is possible that natural sympatric areas exist, none have been identified so far, and the extent and processes of interspecific hybridization in the wild remains unknown [54].

### 3.1. Number of Species in the Genus Phoenix

The number of recognized *Phoenix* species has fluctuated over the years. In an early monograph (Beccari 1890, cited by Barrow, S.A. [5]) ten species were described, while in the latest monograph 13 were recognized [5], see Table 3. An additional species, *P. atlantica*, was later delimited as a distinct species based on microsatellite profiles [48], bringing the current number of *Phoenix* species up to 14 [45]. Nevertheless, a recent genomic study failed to differentiate *P. atlantica* from *P. dactylifera* [33] and its status as a separate species remains to be investigated. In addition, two new species were described: *Phoenix iberica* and *Phoenix chevalieri* [49]. Endemic to Southern Spain, they would grow naturally in barrancos (intermittent streams) and in ravines of the Chicamo River (Murcia province) and would be cultivated in Elche and Fortuna (Alicante Province). However, these are considered by some to be synonyms of *P. dactylifera* [47]. With the recent availability of DNA barcoding methods [50,55], it is now possible to study species delimitation in the genus *Phoenix*, a subject that may advance current understanding of the origins of cultivated date palm.

### 3.2. Phylogenetic Relationships in The Genus Phoenix

The phylogeny of the genus is still unknown. Loci traditionally used in phylogenetic inference are largely uninformative in *Phoenix*. In the last monograph, the combination of morphologic, anatomic, and molecular markers failed to elucidate the relationships of the *Phoenix* species [5]. A clade comprising *P. dactylifera*, *P. sylvestris*, and *P. theophrasti* (the date palm group) was supported by combined analysis of morphological, anatomical, and 5S spacer sequence data. However, a study based on nuclear microsatellites and a chloroplastic minisatellites did not support the monophyly of these species [50]. A study based on chloroplastic sequences identified *P. atlantica* and *P. sylvestris* as date palm sister species and a larger clade comprises these three species along with *P. theophrasti*, *P. canariensis*, and *Phoenix rupicola* [13]. The poor resolution and conflicting relationships reported have left the phylogeny of the members of this genus very much in question. Although short-read sequencing provides important clues to species relationships, a definitive treatment would benefit from whole genome assemblies of all members of the *Phoenix* genus.

## 4. The Wild Ancestor of the Date Palm

The difficulty in distinguishing *Phoenix* species and particularly identifying the sister species of the date palm has hampered the research into date palm origins. Many hypotheses were proposed as to the wild progenitor of the cultivated date palm (see a past paper [51] for review). Some authors have suggested that cultivated date palm derives from wild *P. dactylifera*, now found in small relictual populations [56], while others have stipulated that it derived from another *Phoenix* species with *P. sylvestris* being the most cited, however *P. canariensis*, *P. atlantica*, and *P. reclinata* were also proposed. A last hypothesis posits that the cultivated date palm is a hybrid between two or more *Phoenix* species [51]. In 2010, a nuclear and chloroplastic microsatellite analysis showed that the date palm allelic profile was highly divergent from the other *Phoenix* species [50]. The authors concluded that this crop most probably originate from wild population of the same species, *P. dactylifera*, rather than from another *Phoenix* species.

### 4.1. Identifying Wild Phoenix dactylifera Population

**Figure 2:**
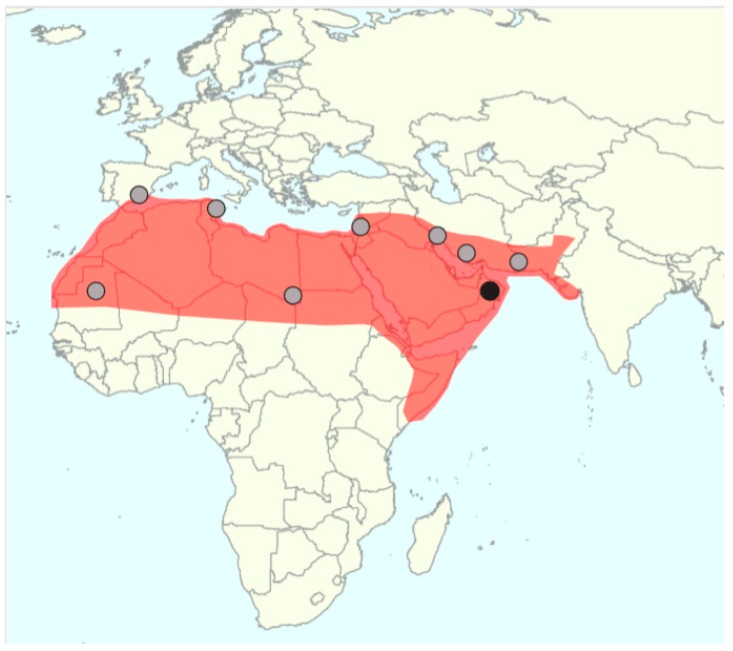
Historical distribution of the date palm (red) with the location of wild date palm populations (black dot) [33] and spontaneous date palm populations of debatable status (grey dots).

The identification of truly wild date palm populations is extremely difficult and until recently it was even doubtful whether wild date palms still exist [5]. Many naturally growing populations have been reported, Figure 2. 

**Figure 3:**
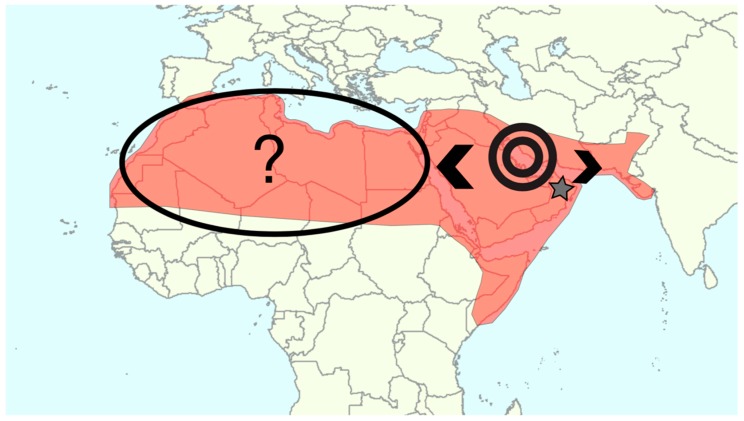
Domestication scenario for the date palm (historical distribution in red) based on the latest lines of evidence in archaeology and genetics. Domestication occurred in the Gulf region (black circles), probably during the 4th millennium BCE from wild populations of *P. dactylifera* of which relict populations were found in Oman (grey star). It was followed by diffusion toward the East and the West (black arrows). African date palms nevertheless appear very distinct and more diverse than Middle Eastern cultivars, involving that an unknown source contributed to this population. The source population, the exact timing and the geographic context of this event remain to be elucidated.

However abandoned, formerly cultivated individuals may survive without human intervention and a population can arise from seeds thrown away in a favorable environment (self-sown date palms). These uncultivated populations are not ancestral to the cultivated genepool, but derive from cultivated populations and, are therefore, feral. For a long time, it was unknown how to distinguish feral from wild date palms and claims of truly wild date palms could not be substantiated. Scientists agree that wild date palms must have smaller, less pulpy, or even nonpalatable fruit compared to cultivated date palms but this may also be the case in feral date palms and even in seedlings from cultivated date palms [57,58]. A new quantitative approach was thus proposed [59] with the quantification of seed shape through geometric morphometrics, a method that has already proven its utility in olives and grapes [60,61]. With a comprehensive analysis of seeds of the genus *Phoenix*, Gros-Balthazard et al. [62] indeed showed that seeds are smaller and round in wild *Phoenix* species while in cultivated and feral date palms they are more elongated. Uncultivated date palms sampled in Oman showed seeds that resemble those found in wild *Phoenix* thus raising the hypothesis that they were wild [60,62]. A microsatellite study of ~100 uncultivated date palms from Oman further demonstrated that their diversity was larger than that found in ~200 cultivars from the Middle East, in accordance with the hypothesis of a genetic bottleneck (i.e., a reduction of diversity due to the fact that only a subset of the wild populations is brought into cultivation) [33]. Whole-genome sequences of three of these putative wild date palms compared to 17 cultivated genomes further supported higher diversity in these samples. A Maximum Likelihood phylogenetic tree suggested that these samples are basal to the Middle Eastern cultivated genepool thus supporting their status as ancestral wild date palms [33], see Figure 3. This discovery opens exciting perspectives and motivates the study of other uncultivated populations that warrant verification, see Figure 2. Additionally, the wild gene pool provides genomic resources for breeding as wild date palms may carry desirable traits for improving cultivated date palms through, for instance, disease resistance.

### 4.2. Consequences of Domestication

Cultivated and wild date palms differ from both a morphologic and genetic point of view. The Middle Eastern cultivated gene pool has retained approximately 80% of the variation found in the Omani wild date palms [33]. This reduction in diversity is consistent with what has previously been reported for other perennials [63]. Their seeds became elongated, probably in relation to a fruit size increase [33,62].

## 5. The Geography of the Domestication

### 5.1. What is the Original Date Palm Distribution?

The natural distribution of date palm is unknown. The historical area of cultivation is North Africa and the Middle East but it is possible that its distribution has been extended far beyond its original growing area [5]. Botanical remains predating agriculture have been found in this region. First, pollen grains were discovered in Shanidar cave, in layers dating back to 50,000 to 33,000 before present (BP) in Northern Iraq [64], and second, charcoal was recovered in Ohalo II, Israel dated at 19,000 BP [65]. It has thus been proposed that the natural distribution of the date palm is found in the Middle East [57], although these sole remains cannot rule out the possibility of a larger distribution. The identification of a wild population of date palm in Oman provides additional evidence of an ancestral population in this region [33]. As stated before, several populations, see Figure 2, or putative species found in the West of the Mediterranean basin (*P. atlantica* in Cape Verde and *P. iberica* and *P. chevalieri* in Spain, Table 3) could be wild date palms, which would drastically expand the assumed natural range of date palms. 

### 5.2. Domestication Center(s)

The oldest records of cultivated date palms are found in the Gulf region, while it appears that phoeniciculture arose later in the archaeobotanical record of North Africa [8]. Based on archaeological evidence, it therefore seems that the date palm originates in the Gulf region. But recent molecular studies based on nuclear microsatellite data have demonstrated the existence of two distinct date palm gene pools in Africa and in the Middle East. Similar results were found using chloroplastic data [13,33,66] and Y-type chromosomal regions [67,68]. Whole genome resequencing data also support a highly differentiated African and Middle Eastern date palms although admixed cultivars found in Egypt and Sudan suggest that this area may constitute a zone of secondary contact between the two gene pools [32,33]. Furthermore, these studies reported higher diversity in African date palms thus challenging the classic view of a domestication event in the Gulf followed by diffusion and a founder event bottleneck in North Africa. 

The hypothesis of another independent domestication event in Africa was tested by Gros-Balthazard et al. [33]. Using 19 genomes of *P. dactylifera*, *P. sylvestris*, and *P. atlantica* they inferred a population graph of population splits and mixtures that indicated that the wild populations of Oman were basal to both cultivated gene pools. African date palms were nevertheless inferred to have received a third of their genomic makeup from an unsampled source. This finding was further confirmed by inferring the demographic parameters of a flexible admixture model. This indicated a contribution of 82% of the Middle Eastern cultivars to the genome of African date palms corroborating the occurrence of a major domestication event in the Eastern part of the distribution followed by a diffusion to the West and subsequent introgression—the stable transfer of alleles across species boundaries—from an unknown source population, see Figure 3.

### 5.3. Diffusion and Interspecific Hybridization

The geographic range of date palm includes zones of sympatry with other members of the *Phoenix* genus, Figure 1. Evidence of interspecific hybridization among *Phoenix* species in anthropogenic contexts (see above) suggests that members of this genus show broad reproductive compatibility [51]. However, until recently, there has been little or no evidence that introgressive hybridization has played a significant role in the evolution of the *Phoenix* genus. Gros-Balthazard et al. [33] found no evidence of admixture between *P. sylvestris* and *P. dactylifera*, even among Indian samples collected where both species occur. Whether the domestication history of date palm included introgressive hybridization from other wild species, Figure 2 and Table 3, and whether it is common throughout the *Phoenix* genus are currently unknown.

## 6. Domestication and Diversification Genes

In date palm, the domestication syndrome is limited primarily to changes in the size and shape of seeds and fruits [33]. At present, nothing is known about genetic basis for these traits in date palm. Recently, however, the genetic basis of a diversification trait—the red/yellow fruit color polymorphism—has been identified. Hazzouri et al. [32] identified the ortholog to the oil palm Virescens gene, an R2-R3 Myb transcription factor that controls the fruit color polymorphism in this distant relative of date palm. Hazzouri et al. further reported that Virescens is truncated in the date palm genome assembly and that all varieties in their study with yellow (or yellow-orange) fruits have a Copia-like retrotransposon in exon 3 of this gene that introduces a premature stop codon. By contrast red-fruit varieties were homozygous for a wild type copy of the gene. A statistical association between the Copia-insertion genotype and fruit color not only supported Virescens as controlling this trait in date palm, but also suggested that this mutation acts as a dominant negative as it does in oil palm. 

The identification of the domestication and diversification genes through genome-wide association studies will greatly enrich understanding of the genetic architecture of date palm domestication. This may provide clues as to the nature, timing, and geographic context of artificial selection on these traits.

## 7. Conclusions and Prospects

In this paper we reviewed the recent advances in the field of date palm genomics and provide the latest results on the domestication and diversification history of the date palm. Approximately 100 date palm and wild relative genomes are presently available in GenBank, providing new opportunities to study this species. These genomic data have provided answers to longstanding questions about date palm origins including identification of wild populations that served as the progenitor to cultivated date palm and also raised questions about the origins of African date palms. In fact, genomic data point out that date palm domestication was probably significantly more complex than once imagined. 

Many questions remain unresolved including the timing of domestication, the origins of African date palms and the genes and selective mechanisms responsible for the enormous diversity observed in date palm varieties. Improved genomic technologies along with better algorithms to study population genomics and the basis of phenotypic traits make this a particularly exciting time to study the origins of the date palm.

## Figures and Tables

**Table 1:** List of nuclear and organellar assembly of date palm in September 2018.

Genome	Cultivar	GenBank Reference Number	Size	Number of Scaffolds	N50	Reference
**Nuclear**	Khalas	GCA_000181215.2	~381 Mb	57,277	30,480	Al-Dous et al., 2011 [19]
**Nuclear**	Khalas	GCA_000413155.1	~558 Mb	82,354	329,900	Al-Mssallem et al., 2013 [20]
**Chloroplastic**	Khalas	NC_013991.2	158,462 bp	/	/	Yang et al., 2010 [21]
**Chloroplastic**	Aseel	FJ212316.3	158,458 bp	/	/	Khan et al., 2012 [22]
**Chloroplastic**	Khanezi	/	158,211 bp	/	/	Khan et al., 2018 [23]
**Chloroplastic**	Naghal	/	158,210 bp	/	/	Khan et al., 2018 [23]
**Mitochondrial**	Khalas	NC_016740	715,001 bp	/	/	Fang et al., 2012 [24]
**Mitochondrial**	*unknown*	MG257490.1		/	/	/
**Mitochondrial**	Khanezi	MH176159.1		/	/	/

**Table 2:** Comprehensive list of whole-genome resequencing studies of date palms and wild relatives in September 2018. # accession is the number of *Phoenix* spp. individuals included while (# new) refers to the number of new sequences generated for the study.

Reference	# Accession (# New)	Summary of Major Findings
Al-Dous et al., 2011 [19]	9 (9) cultivated date palms	First genome assembly of the date palm genome (cultivar Khalas). The paper further focuses on sex determination, providing the evidence that the date palm employs an XY system of gender inheritance.
Al-Mssallem et al., 2013 [20]	11 (4) cultivated date palms	Improved genome assembly of the date palm genome (cultivar Khalas) and study of genetic diversity among a few cultivars. Functional genes involved in stress resistance and sugar metabolism were brought to light.
Hazzouri et al., 2015 [32]	62 (62) cultivated date palms	Resequencing study of 62 cultivars from North Africa and the Middle East providing evidence for a large differentiation between these two gene pools. A larger diversity in North African date palms is noted, challenging the classic scenario stating that they derive from Middle Eastern cultivars. The orthologue of the oil palm Virescens gene was linked to color polymorphism (red/yellow) in dates.
Gros-Balthazard et al., 2017 [33]	16 (2) date palms 3 (3) wild date palms 1 (1) *Phoenix sylvestris* 1 (1) *Phoenix atlantica*	Candidate wild date palms growing in Oman were hypothesized based on seed morphometric features and diversity analyses (microsatellite data). Further whole-genome analyses, including structure, diversity, and modeling, demonstrated that they are ancestral, leading to the first report of wild date palms. African date palms were shown to mostly derive from Middle Eastern cultivars although an unknown source of variability was noted.
Torres et al., 2018 [34]	15 female and 13 male individuals representing all 14 species (no male *Phoenix pusilla* could be identified for sequencing)	Whole-genome sequencing of males and females from all *Phoenix* species allowed the identification of male-specific sequences. The sex-determination region was further sequenced using long read technologies and annotated. Four genes were identified and their analysis supported a two-mutation model for the evolution of dioecy in *Phoenix*.

**Table 3:** List of *Phoenix* species based on the latest monograph of the genus [5]. Detailed information on their status, morphology, uses, and distribution may be found in a previous paper [5] and on Palmweb [47]. Additional references are indicated for species described after the publication of the monograph or when further information was recently reported.

Species	Current Status	Distribution
*Phoenix acaulis*	Recognized species	Southern Asia
*Phoenix andamanensis*	Recognized species	Andaman and Nicobar Islands
*Phoenix atlantica* [48]	Recognized species but status warrant verification following a genomic study that failed to differentiate it from African date palms [33]	Cape Verde (see Figure 1)
*Phoenix caespitosa*	Recognized species	Southern Arabia and Horn of Africa (see Figure 1)
*Phoenix canariensis*	Recognized species	Canary Islands (see Figure 1)
*Phoenix chevalierii* [49]	Unrecognized species	Southern Spain
*Phoenix dactylifera*	Recognized species	Southern Spain, North Africa, the Middle East, Pakistan, and Northwestern India (see Figure 1) Recently introduced in many locations including California and China
*Phoenix iberica* [49]	Unrecognized species	Southern Spain
*Phoenix loureiroi*	Recognized species	Southern Asia
*Phoenix paludosa*	Recognized species	Southern Asia
*Phoenix pusilla*	Recognized species	Indian subcontinent
*Phoenix reclinata*	Recognized species	Sub-Saharan Africa (see Figure 1)
*Phoenix roebelenii*	Recognized species	Southeast Asia
*Phoenix rupicola*	Recognized species	Foothills of the Himalayas
*Phoenix sylvestris*	Recognized species	Indian subcontinent (see Figure 1)
*Phoenix theophrasti*	Recognized species	Crete and coastal Turkey (see Figure 1)
